# Modeling the Influence of Oil Film, Position and Orientation Parameters on the Accuracy of a Laser Triangulation Probe

**DOI:** 10.3390/s19081844

**Published:** 2019-04-18

**Authors:** Chengxing Wu, Baijin Chen, Chunsheng Ye, Xiaopeng Yan

**Affiliations:** State Key Laboratory of Material Processing and Die & Mould Technology, Huazhong University of Science and Technology, Wuhan 430074, China; wcx0298@hust.edu.cn (C.W.); csye@hust.edu.cn (C.Y.); yanxp@hust.edu.cn (X.Y.)

**Keywords:** laser triangulation probe, measurement accuracy, oil film, position and orientation parameters, error modeling

## Abstract

The laser triangulation probe conveniently obtains surface topography data of a measured target. However, compared to the touch probe, its reliability and accuracy can be negatively affected by various factors associated with the object being measured and the probe itself. In this paper, to identify potential compensation strategies to improve the accuracy of depth measurement for laser triangulation probe, the measuring errors caused by an oil film on the measured surface, and the probe’s position and orientation parameters with respect to the measuring object (including scan depth, incident angle, and azimuth angle), were studied. A theoretical model based on the geometrical optics, and an empirical model from the error evaluations, were established to quantitatively characterize the error influence of oil film and probe’s parameters, respectively. We also investigated the influence pattern of different filtering methods with several comparison experiments. The verification procedures, measuring both a free-form surface (chevron-corrugated plate) and a gauge block covered with an oil film, demonstrate that these models and measurement suggestions are viable methods for predicting theoretical error and can be used as compensation references to improve the accuracy of depth measurement to the laser triangulation probe.

## 1. Introduction

As a result of the merger of technological advances in both the optical and sensor industries [1,2,3], the laser triangulation probe has gradually replaced the contact probe in various fields, and it is usually characterized as a precise measurement device to effectively acquire the depth information of an object. Therefore, it is widely applied in the fields of product design and manufacturing and industrial quality inspection [4,5,6,7]. However, compared with the traditional contact probe, although the triangulation probe on the market can meet the requirements of time limitation and untouched on-line measurement for digitizing techniques, the measuring accuracy and reliability are easily influenced by various factors [8], because of its overly reliance on the measurement requirements of both the consistency of object-surface scattering light intensity and a high level of consistency between the laser optical axis and the normal vector at the measuring point [9,10]. However, there still exist some differences between the theoretical requirements and practical operations in the field of industrial measurements because the measured surface is almost inevitably covered with residual greasy dirt or other substances, and the measuring target is usually a complicated non-uniform shape. In general, most of these differences [11] are directly influenced by the surface optical characteristics and/or the probe’s position and orientation parameters with respect to the measuring object; such influence factors can be further considered as the primary sources of measurement error [12,13]. Therefore, for taking advantage of this optical technology fully on the depth measurement, it is necessary to have a best control of these error factors.

Most studies have aimed to minimize the errors in the measurement accuracy caused by the surface characteristics, mainly by focusing on the measured object itself. The factors considered include reflectivity [14,15,16], roughness [17], and color [18,19]. Few studies have focused on the presence of additional substances on the measured surface, such as an oil film, rust, or a paint coating.

When investigating the influence of a probe’s position and orientation parameters, for example scan depth, incident angle, and azimuth angle, research has mainly been performed using modeling analyses. However, the majority of these studies separately investigated the influence of each parameter. For instance, as the inclined plane can affect the laser beam and cause the migration of the imaging on the charged-coupled device (CCD), Dong et al. [20] analyzed the migration regularity of the imaging spot and reestablished a mathematical model to calculate the measurement displacement of the laser triangulation probe. Li et al. [21] and Sun et al. [22] proposed a compensation model related to the incident angle based on the geometrical optics of the measurement for the inclined plane, as they thought that the incident angle has a greater impact on the measuring accuracy than the incident angle and azimuth angle. However, this approximate treatment may result in accumulative error during modeling parameter optimization. Feng et al. [23] experimentally characterized the influence pattern of the scan depth and incident angle and proposed an empirical model related to those parameters to perform software compensation. In Isheil et al. [24], a global error model associated with the scan depth, incident angle and orientation angle was built to reduce their overall impact of measuring errors on the system accuracy. However, as they focused on the research related to the line-scanning sensors, the proposed correction strategies ignore the uncertainties caused by the inconsistency of light intensity along the scanned line.

The objective of this study was to identify potential compensation models that reduce the influence of error factors on the accuracy of depth measurement to a laser triangulation probe. Our main aims were as follows: (1) to characterize the errors caused by an oil film on the measured surface, analyses combined with the laser triangulation principle and refractive optics were completed to establish a theoretical error model; and (2) to consider the integrated influence of the probe’s position and orientation parameters (including scan depth, incident angle, and azimuth angle) on the procedure of free-form surface measurement, and to establish an empirical error model according to the regression analysis of the evaluation results. By performing several comparison experiments, the influence pattern of filter methods was also discussed. Finally, the effectiveness of the compensation strategies was verified by comparing the modeling results and the experimental data or the standard computer automated design (CAD) data.

## 2. Measurement Principle

**Figure 1:**
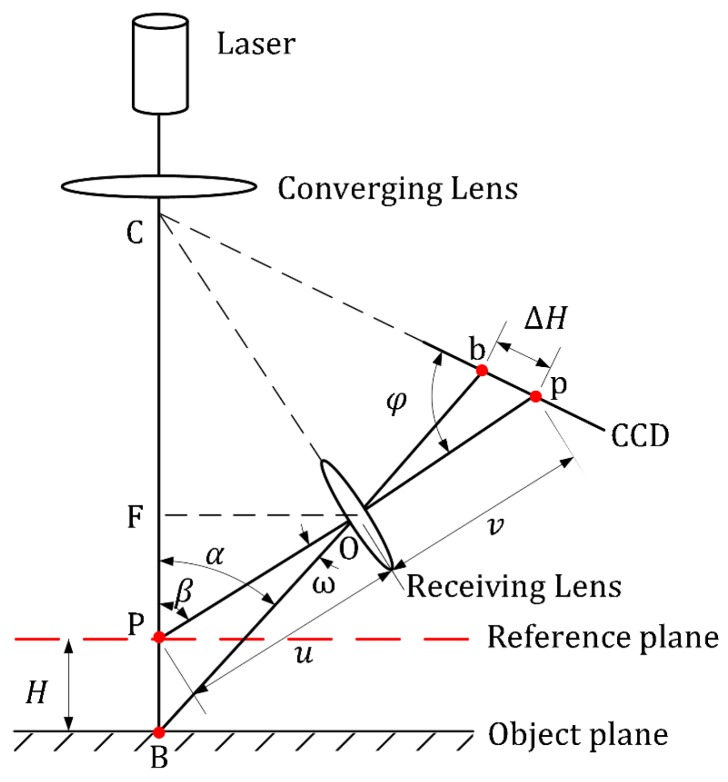
A schematic diagram of a typical direct laser triangulation.

The optical triangulation principle can be divided into two categories: the direct type and the oblique type. Compared with the oblique type, the direct type has output wavelength stability, high light intensity concentration, and a small laser spot diameter. Hence, the direct triangulation principle is more suitable for the depth measurement of objects with good light diffusion properties. As Figure 1 shows, the focused laser beam emitted from the laser is projected onto the object’s surface. Part of the scattering light is projected onto the charged-coupled device (CCD) detector to form a laser light imaging spot caused by the diffuse reflection. According to the geometrical relations and imaging laws, the theoretical measuring displacement *H* is determined by calculating the transverse offset’s (ΔH) image center points on the CCD. Their relationship can be expressed as:

(1) $$H\;=\;\frac{u\Delta H\sin\varphi }{v\sin\beta \;-\;\Delta H\sin\left(\beta \;+\;\varphi \right)},$$

where *β*, *φ*, *u*, *v*, are the design parameters of the laser triangulation probe. *β* is the angle between the axis of the laser beam and the receiving lens, *φ* is the angle between the CCD detector and the optic axis of the receiving lens, and *u* and *v* are the object distance and the image distance of the receiving lens, respectively.

To obtain a clear image of a moving target on the CCD detector, the design parameters (including *u*, *v*, *β*, *φ*) should satisfy the Scheimpflug condition [25], that is:

(2) utanβ = vtanφ

## 3. Influence of An Oil Film on Depth Measurement

In the field of industrial measurement, the surface of the measuring part is unavoidably covered with non-identical substances, such as water, oil, dust, paint coating, and other additional elements, which may change the original optical characteristics of measuring surface and thus reduce the accuracy of the depth measurement to an object. In this section, on the basis of the analyses of both refractive optics and triangulation principle, we establish a theoretical error model to characterize the influence of an oil film.

### Modeling Analysis

**Figure 2:**
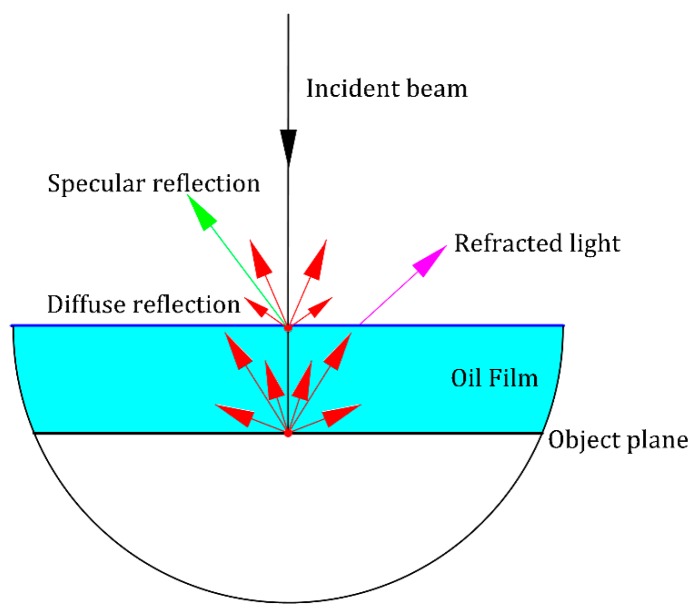
Local magnification of the change for incident light intensity.

Based on the laser triangulation principle, the precise geometrical relations of the light sources and a good diffuse reflection property of the measuring surface are the precondition of achieving high-precision optical measurement. As illustrated in Figure 2, when the incident laser beam travels through a medium with a different refractive index, such as an oil film, the refraction occurring at the interface between the oil film and air can lead to the change of the intensity distribution of laser reflected from the measured surface, and further result in the decrease of the ratio of the intensity of diffuse reflection light $L_{D}$ [18]. The ratio ε can be mathematically expressed as:

(3) $$\varepsilon \;=\;\frac{L_{D}}{L_{D}\;+\;L_{S}\;+\;L_{R}},$$

where $L_{S}$, and $L_{R}$ donate the intensities of specular reflection and refraction, respectively. The sum of those light intensity ($L_{D},\;\;L_{S},\;\;L_{R}$) is approximately equal to the initial energy of the incident light.

**Figure 3:**
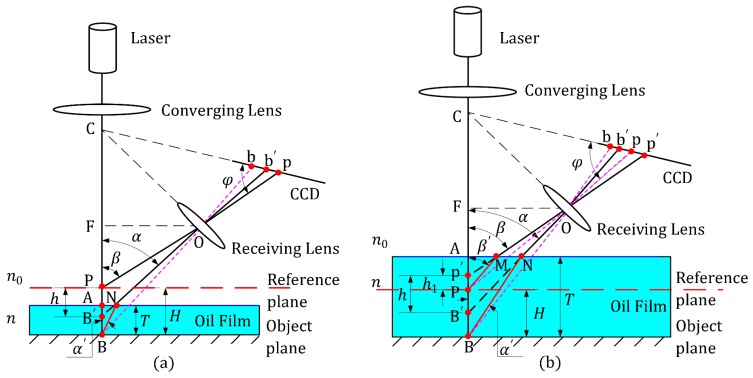
A schematic optical path under refraction conditions. The reference plane lies (**a**) above the oil film and (**b**) in the oil film.

The variation of the ratio about the intensity of reflection on the measuring surface can result in the magnification of the imaging lens to vary unevenly within its field of view. This directly causes a change in the calibration distance between the CCD imaging plane and the main energy plane of the reflected light. Unfortunately, this distortion to the optics may introduce errors into the measurement results. The following equation can be used to calculate the measurement error *e* caused by the oil film:

(4) e = H − h,

where *h* denotes the practical measurement result, as shown in Figure 3.

The reference plane of the optical probe is virtual (as shown in Figure 3), and can be adjusted arbitrarily in theory. In this section, the influence of the oil film is discussed according to the relative position between the reference plane and the upper surface of the oil film.

In the first case (Figure 3a), the incident laser beam is focalized through the converging lens and is perpendicularly incident to point B on the object plane, P is the theoretical reference point about the measuring system. As a result of the differences in the refractive index, the imaging spot on the CCD for point $B^{\prime }$ deviates from the theoretical position b to the practical imaging position $b^{\prime }$. From another perspective, $b^{\prime }$ can be considered the imaging spot of $B^{\prime }$, where $B^{\prime }$ is the intersection point of the laser optical axis with the reverse extension of its refraction ray ${\mathrm{Nb}}^{\prime }$, N is the intersection point of the scattered light of BN with the upper surface of the oil film. Finally, in accordance with the light refraction principle, the following equation is possible:

(5) $$n\sin\alpha^{\prime }\;=\;n_{0}\sin\alpha ,$$

where $\alpha^{\prime }$ is the angle between the laser optical axis and the scattered light of BN.

In $\Delta B^{\prime }\mathrm{FO}$:

(6) $$\sin\alpha \;=\;\frac{u\sin\beta }{\sqrt{{\left(u\sin\beta \right)}^{2}\;+\;{\left(u\cos\beta \;+\;h\right)}^{2}}},$$

Similarly, in ΔABN and $\Delta {\mathrm{AB}}^{\prime }N$:

(7) $$\mathrm{AN}\;=\;\left(h\;-\;H\;-\;T\right)\tan\alpha \;=\;T\tan\alpha^{\prime },$$

Substituting Equations (5) and (6) into Equation (7), the relationship between the theoretical measuring value *H* and the practical *h* can be obtained as:

(8) $$H\;=h\;+\;T\left(1\;-\;\frac{n_{0}\left(u\cos\beta \;+\;h\right)}{\sqrt{\left(n^{2}\;-\;n_{0}^{2}\right){\left(u\sin\beta \right)}^{2}\;+\;n^{2}{\left(u\cos\beta \;+\;h\right)}^{2}}}\right),$$

For the second case (Figure 3b), Based on our analysis of the first case, both the spots $b^{\prime }$ and $p^{\prime }$, which are corresponding to the migration results of the imaging spots b and p on the CCD, can be regarded as the imaging spots of $B^{\prime }$ and $P^{\prime }$, respectively. Where $P^{\prime }$ is the intersection point of the laser optical axis with the reverse extension of refraction ray ${\mathrm{Mp}}^{\prime }$, and it also stands for the actual reference point when there exists an oil film, M is the intersection point of the scattered light of PM with the upper surface of the oil film. In this situation, the same geometrical optics relationships exist:

(9) $$n\sin\beta^{\prime }\;=\;n_{0}\sin\beta ,$$

where $\beta^{\prime }$ is the angle between the laser optical axis and the scattered light of PM.

In ΔAPM and $\Delta {\mathrm{AP}}^{\prime }M$, ΔABN and $\Delta {\mathrm{AB}}^{\prime }N$:

(10) $$\mathrm{AM}\;=\;\left(T\;-\;H\;-\;h_{1}\right)\tan\beta^{\prime }\;=\;\left(T\;-\;H\right)\tan\beta ,$$

(11) $$\mathrm{AN}\;=\;T\tan\alpha^{\prime }\;=\;\left(T\;-\;H\;+\;h\;-\;h_{1}\right)\tan\alpha ,$$

where $h_{1}$ is the distance between the theoretical reference point P and the actual $P^{\prime }$.

By joining Equations (5), (6), (9)–(11), we determine that *H* can be expressed as:

(12) $$H\;=\;\frac{\sqrt{n^{2}\;-\;{\left(n_{0}\sin\beta \right)}^{2}}}{n_{0}\cos\beta }\left[h\;+\;T\left(\frac{n_{0}\cos\beta }{\sqrt{n^{2}\;-\;{\left(n_{0}\sin\beta \right)}^{2}}}\;-\;\frac{n_{0}\left(u\cos\beta \;+\;h\right)}{\sqrt{\left(n^{2}\;-\;n_{0}^{2}\right){\left(u\sin\beta \right)}^{2}\;+\;n^{2}{\left(u\cos\beta \;+\;h\right)}^{2}}}\right)\right],$$

As a result, the measuring error *e* caused by the oil film can be modeled as:

(13) $$e\;=\;\{\begin{array}{cc} T\left(1\;-\;\frac{n_{0}\left(u\cos\beta \;+\;h\right)}{\sqrt{\left(n^{2}\;-\;n_{0}^{2}\right){\left(u\sin\beta \right)}^{2}\;+\;n^{2}{\left(u\cos\beta \;+\;h\right)}^{2}}}\right) & \left(\mathrm{case}1\right)\\ \frac{\sqrt{n^{2}\;-\;{\left(n_{0}\sin\beta \right)}^{2}}}{n_{0}\cos\beta }\left[h\;+\;T\left(\frac{n_{0}\cos\beta }{\sqrt{n^{2}\;-\;{\left(n_{0}\sin\beta \right)}^{2}}}\;-\;\frac{n_{0}\left(u\cos\beta \;+\;h\right)}{\sqrt{\left(n^{2}\;-\;n_{0}^{2}\right){\left(u\sin\beta \right)}^{2}\;+\;n^{2}{\left(u\cos\beta \;+\;h\right)}^{2}}}\right)\right]\;-\;h & \left(\mathrm{case}2\right) \end{array},$$

where $n_{0}$ and n are the refractive indexes of the air and the oil film, respectively. $n_{0}$ can be approximately taken as an integer constant 1, and *T* is the thickness of oil film.

In the established model in Equation (13), the variables n and *T* can be obtained with an instrument in advance, and the measured result *h* can be read from the measuring system. Using the derived theoretical model, a theoretical error can be predicted in cases where an oily substance exists on the surface of the measurement target. The measuring error generally increases with the characteristic parameters of the oil film, such as thickness *T* and refractive index n. In the practical depth measurement, measuring errors caused by oil film are mostly produce in the case 1, as the oil film on the measured surface is usually relatively shallow.

## 4. Influence of Probe’s Position and Orientation Parameters on Depth Measurement

**Figure 4:**
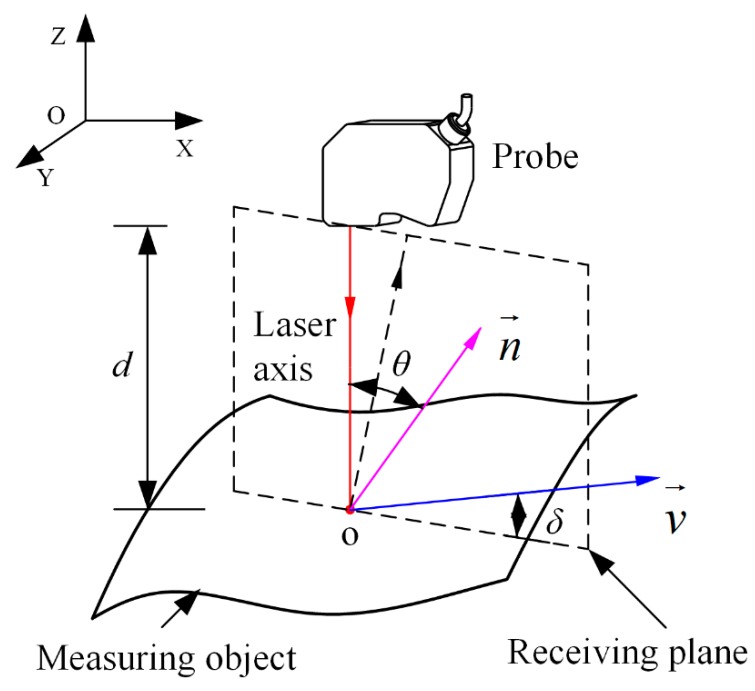
The definition of probe’s position and orientation parameters.

One of the fundamental assumptions of the laser triangulation principle with respect to ensuring measuring accuracy is that the incident light is always aligned with the normal vector at each measurement point. However, during the depth measurement of a free-form surface, the impact of a non-uniform distribution of curvatures at measurement points can result in variations of the probe’s position and orientation parameters, such as the scan depth, incident angle, and azimuth angle. Therefore, if the probe’s control system still adopts a primitive calibration curve to calculate the measurement displacement, the accuracy of the measurement system will be impaired. In this section, by simulating the measurement of the free-form surface with an inclined model, the integrated influence of the probe’s position and orientation parameters is analyzed with experimental evaluation. The probe’s parameters, which defined the relative position and orientation between the probe and the measuring object, including the scan depth *d*, incident angle *θ*, and azimuth angle *δ*, can be seen in Figure 4:

(1) *d* stands for the distance between the measurement point and the probe;
(2) *θ* is the angle between the optical axis of probe’s converging lens and the normal vector $\vec{n}$ at the measurement point;
(3) *δ* is the angle between the probe’s moving direction $\vec{v}$ and probe’s receiving plane. Where the receiving plane is composed of the incident beam and the optical axis of the receiving lens.

### 4.1. Error Evaluation

**Figure 5:**
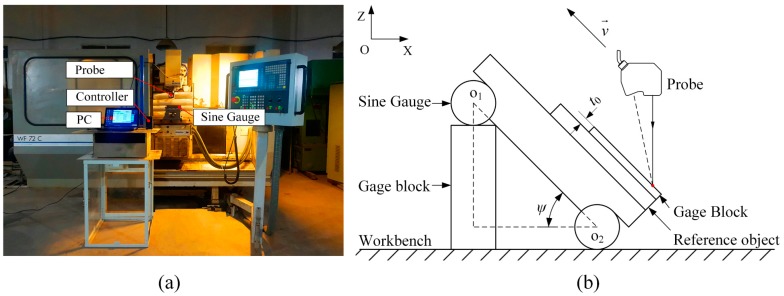
(**a**) Overview of the evaluation system; (**b**) an inclined evaluation model.

To evaluate the errors under various probe’s parameters (*d*, *θ*, and *δ*), the evaluation system based on the inclined model is shown in Figure 5. The system was composed of a laser displacement sensor (one-dimensional; 1D), a sine gauge, a set of standard gauge blocks, a four-axis MIKRON type WF 72 C of computer numerical control (CNC) milling machine (MIKRON, Agno, Switzerland), and a personal computer (PC). Among that, a data acquisition software LK-Navigator 2 (KEYENCE, Osaka, Japan) was installed in the PC equipped with a 2.5 GHz Intel Core i5-3210M CPU, 64-bit Windows 10 operating system and 8 GB memory. The sensor was composed of a LK-G5000 controller (KEYENCE, Osaka, Japan) and a LK-H080 probe (KEYENCE, Osaka, Japan) (see Table 1 for its main configuration parameters), and all measurement data stored in the controller were transferred to the PC via USB communication ports.

The probe was mounted on the spindle of CNC machine (MIKRON, Agno, Switzerland), and each axis of the CNC milling machine was 0.01 mm. Further, the synchronous motion of the CNC machine in the X and Z directions ensures that the probe always moves parallel to the measured surface at a uniform velocity. For the measurement system, a standard ball driven by a rotary worktable and rotated around the Z-axis was used to conduct the simplified calibration procedure. By calculating the measurement uncertainty of the relative center of the ball, the system accuracy was eventually confirmed to be 52 μm.

In our evaluation procedure, the spindle of the CNC machine guides the probe’s vertical movement to adjust the scan depth *d*, the incident angle *θ* is changed by adjusting the height of the standard combinatorial gauge using Pythagorean theorem, and the azimuth angle *δ* can be varied by changing the installation angle of the probe with respect to the spindle. The scan depth *d* ranged from 67 mm to 93 mm in increments of 2 mm, the incident angle *θ* varied in the range of 0° to 60° in increments of 5°, and several conditions for the azimuth angle *δ* (0°, 30°, 60°, 90°) were studied.

In Figure 5b, both the gauge block and the reference object were measured. $t_{0}$ was set to the reference thickness of the measuring gauge block, which was obtained by the coordinate measurement machine (CMM). By contrasting the reference value $t_{0}$ with the experimental values, the measurement error $e_{i}$ caused by each group of parameters (*d*, *θ*, and *δ*) was obtained:

(14) $$e_{i}\;=\;\left(md_{i}\;-\;md_{i_{R}}\right)\;/\;\cos\psi \;-\;t_{0},$$

where, after the test was repeated five times, $md_{i}$ and $md_{i\_R}$ are the average results of each test for measuring both the gauge block and reference object, respectively, and *ψ* denotes the inclined angle of the measured model, which is equivalent to the incident angle *θ*.

**Figure 6:**
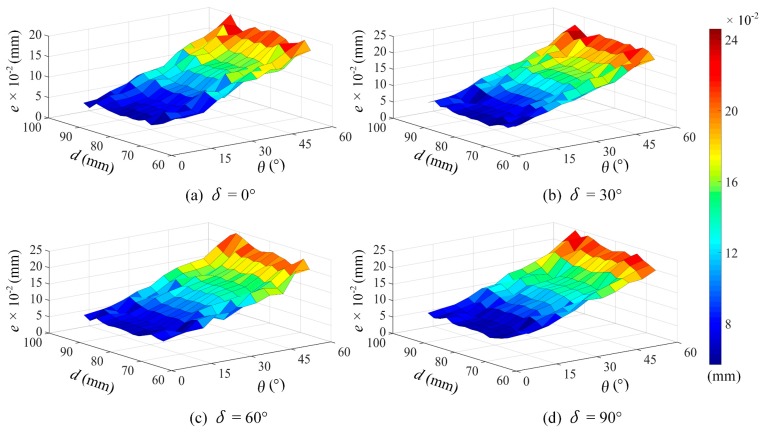
Evaluation results on the inclined model: (**a**–**d**) show the error maps obtained under the conditions of azimuth angle *δ* = 0°, *δ* = 30°, *δ* = 60°, *δ* = 90°, respectively.

After a series of tests, we obtained the error maps related to the various parameters (*d*, *θ*, and *δ*); Figure 6 shows the evaluation results. It can be seen that under the same measuring conditions, the errors increase strongly with the increase in azimuth angle *δ*; and when *δ* is certain, the errors increase with either the increase in scan depth *d* or the incident angle *θ*. However, within the entire measurement range of the laser probe, when *θ* and *δ* are fixed, the changing regularity of the error maps as related to the scan depth *d* is complicated. Conversely, when *d* is in the range of 75 mm to 85 mm, which means that the measuring object is located near the center of probe’s measuring range, the errors is relatively small with more stable variations. This analysis reveals that in this case, the errors caused by the scan depth *d* is relatively small and its influence on measuring accuracy can be approximately ignored.

### 4.2. Modeling Analysis

**Figure 7:**
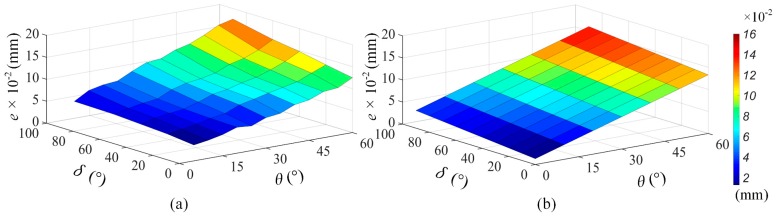
(**a**) Origin error map for the scan depth *d* = 79 mm; (**b**) the fitting form obtained from the empirical model in Equation (15).

Since the overall variation regularity for the errors caused by the various parameters (*d*, *θ*, and *δ*) was not consistent, considering their overall influence on the entire measuring range of the probe was difficult. In the practical application of micro-displacement measurements, the laser data usually vary within a narrow range. Based on the above analysis, the modeling analysis for the overall influence of the probe’s parameters was developed under the conditions of scan depth *d* in the range of 75 mm to 85 mm because the measuring error produced under such conditions can be mainly ascribed to the incident angle *θ* and azimuth angle *δ*. After considering the forms of the error maps, in particular that of Figure 7a for the scan depth *d* = 79 mm, we adopted both the regression method and the least-squares method (LSM) to characterize the relationship between the error and parameters (*θ*, *δ*). Figure 7b provides the modeling results, and the empirical error model can be fitted by a plane model:

(15) e = 0.000035θ + 0.000005δ + 0.013,

which only holds under the condition that the measuring target is located near the center of probe’s measuring range; *θ* and *δ* are within the ranges of 0° to 60° and 0° to 90°, respectively.

To implement the error-compensation procedure for free-form surface depth measurement, the key parameters (*θ*, *δ*) in the established model in Equation (15) should be obtained first. As the incident angle *θ* at each point is closely related to the normal vector of the local set of points, the analysis of the distribution of the point’s normal vector is the first step in identifying the incident angle *θ*. By first identifying the optimal *k*-nearest neighbor for any given measurement point, the normal vector of the tangent plane can be approximately represented as its actual normal vector, obtained using the method of approximating the prototype body by the tangent plane [26].

It was necessary to analyze the variation regularity of the probe’s moving direction and the normal vector of the receiving plane to obtain the azimuth angle *δ*. Since the relative motion of the probe is driven by two parallel mechanisms and moves along a prescribed scanning trajectory curve, its moving direction at each measuring point can be calculated from the vector obtained from the two directions. The receiving plane’s normal vector can be estimated from the movement trajectory of the connection apparatus as the probe is usually fixed onto the connection apparatus by a spindle or something equivalent. Then, the parameters (*θ*, *δ*) were calculated based on their geometrical relationship, as shown in Figure 4.

## 5. Other Error Factors

In the measurement system, to meet the demands of real-time noise elimination and to decline the influence of non-based frequency signal, it is necessary to implement the real-time filtering based on hardware. In this section, we mainly discuss the influence due to the filtering processing by different methods, which is a significant issue in raw data processing for laser triangulation probes.

### Filtering Processing

**Figure 8:**
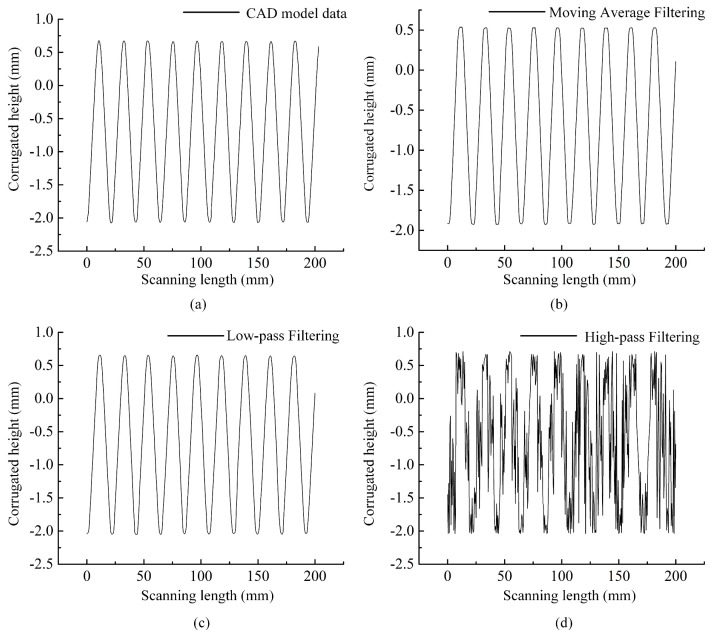
(**a**) CAD model data; (**b**) data processed using the moving average filtering method *N* = 256; (**c**) data processed using the loss-pass filtering method $f_{c}$ = 300 Hz; (**d**) data processed using the high-pass filtering method $f_{c}$ = 300 Hz.

Real- or frequency-space filtering processing have been widely used as a pre-processor for automated data analysis tools [27], because the raw data usually include invalid data caused by the hardware system and/or the measurement environment. The filtering to the raw data can be completed via the open source software or commercial software. In our test, the LK-Navigator 2 software provides several filtering methods such as the moving average filtering, low-pass filtering, and high-pass filtering, each of methods has their own filtering parameters. In this section, based on the multi-section measurement of a chevron-corrugated plate, we used several comparison experiments to investigate whether the application of a digital filter influences the accuracy of free-form surfaces measurement. The parts of one cross-section of the laser data and its corresponding computer automated design (CAD) model data are displayed in Figure 8a–d.

Comparing the measurement data processed by various filtering methods with the CAD data in Figure 8a, when measuring the complex free-form surface, the laser data of the object cannot be effectively obtained using the high-pass filtering method to process the measurement signal (as shown in Figure 8d). Conversely, the moving average filtering method suppresses interference and noise by simply averaging the sampling signals in a certain filtering length, which can be defined as [28]:

(16) $$y\left[n\right]\;=\;\frac{1}{N}\sum_{j=0}^{N-1}x\left[n\;+\;j\right],$$

where y[n] and x[n] are the output signal and the input sampling signal, respectively. *N* is the number of sampling points for averaging. The choice of the number of filtering points or filtering length plays a decisive role on application effect of the moving average filtering method, which is usually selected according to the sampling frequency. As illustrated in Figure 8b (*N* = 256), it characterizes the approximate contours of the measured object. However, the local detail information at the peaks and troughs is different, which directly causes the cross-section contour to vary from a sinusoidal to an approximately trapezoidal curve, eventually leading to the distortion of the measured data. Compared with these techniques, the low-pass filtering method can block signals outside its passband by setting a reasonable cut-off frequency ($f_{c}$) parameter, ultimately achieving non-linearity correction for the aberrance of signals [29].

After several experiments with different $f_{c}$, we found that the change tendency of the measuring signal is less sensitive to variations in $f_{c}$ and sampling frequency. Figure 8c shows that the geometric characteristics of the measured object are well reserved, being the closest to actual CAD model.

Regarding the analysis above, we conclude that filtering processes must be considered an error factor as they directly affect the accuracy of the measured data. Therefore, to further strengthen the anti-jamming capability of the system and reduce the amount of errors originating from the filtering process, the selection of the filtering method and corresponding filtering parameters should comprehensively consider the scope and limitations of these filtering methods, as well as the surface shape of the measuring object.

## 6. Experimental Verification

In this section, to verify the effectiveness of our proposed error models and measuring suggestions, three gauge blocks, which were covered with different oily substances (corresponding refractive index: n1 = 1.448, n2 = 1.461, n3 = 1.475), and a chevron-corrugated plate, are used to simulate the practical measuring situations, which correspond to that there exist the oil film on the measuring surface and the free-form surface measurement, respectively.

### 6.1. Measurement with the Presence of Oil Film

**Figure 9:**
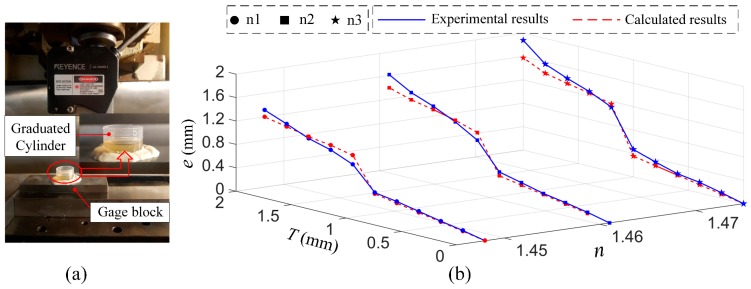
(**a**) The measurement experiment with the presence of an oily substance on the measured object; (**b**) comparative results for both the experiment and model.

As shown in Figure 9a, a section of graduated cylinder was placed on the gauge block. In our test, we utilized a syringe to ensure equal quantities of oil were added at each addition, and the range of the thickness of oil film was 0 mm to 2 mm. Each test was repeated five times, and the thickness of the oil film was increased by 0.2 mm for each test. A total of 150 tests were performed on three oily substances. The measuring error $e_{N\_i}$ for each type of oily substance at different thicknesses can be defined as:

(17) $$e_{N\_i}\;=\;d_{N\_i}\;-\;d_{0},$$

where $d_{N\_i}$ represents the *i*th average measuring result for measuring the *N*th type of oily substance, and $d_{0}$ is the initial measuring value before the addition of any substance.

Figure 9b compares the calculated and experimental errors of different variables with thickness *T* and refractive index n. The three blue solid lines represent the experimental results for oily substances n1, n2, and n3, and their corresponding red dotted lines are the calculated results from the model in Equation (13). The maximum error caused by the oil film is much greater than the system calibration accuracy (52 μm), which indicates that the presence of an oil film is one of the most important sources of error affecting the measurement accuracy of the laser triangulation probe.

Although the maximum percentage deviation between the calculated and experimental results was 20%, which may have been caused by the instability of the measuring environment, the relative consistency of the comparison results demonstrates the usability and validity of the established error compensation model in terms of reducing the influence of the oily film. Among the characteristic parameters of the oil film, the impact of the refractive index n is much greater than that of the thickness *T*. Despite the existence of remarkable local irregularity as the probe’s reference plane approaches the upper surface of the oil film, the measuring error increases approximately in line with the increase in both the thickness *T* and refractive index n of the oil film.

### 6.2. Measurement of the Free-Form Surface

**Figure 10:**
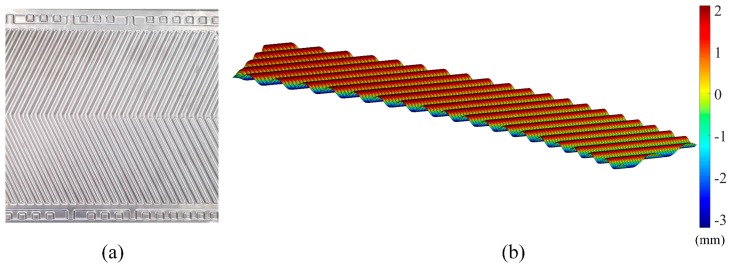
(**a**) The chevron-corrugated plate; (**b**) part of the measurement data.

To simulate the procedure of free-form surface measurement and validate the effectiveness of the empirical error model in Equation (15), a chevron-corrugated plate was used for the analysis, as illustrated in Figure 10a. The original data $P_{o}$ (about 24,000 points, Figure 10b) were acquired via the laser triangulation measuring system using the translation and rotation method; the low-pass filtering method was selected and set to $f_{c}$ = 300 Hz. The measured part was treated to ensure the surface had a homogeneous metallic luster before being placed at the center of probe’s measuring range. Then a few application prerequisites for the empirical compensation model listed in Section 4.2. were satisfied.

On the basis of the model in Equation (15), after obtaining the probe’s parameters (*θ*, *δ*) at each measuring point, the corresponding error at each point was obtained. We then compared the measurement results $P_{c}$ after compensation and the benchmark data $P_{b}$ of the actual CAD model. The quantitative improvement (w) by percentage can be mathematically expressed as:

(18) $$w\;=\;\left|\frac{P_{c}-P_{b}}{P_{o}-P_{b}}\right|,$$

**Figure 11:**
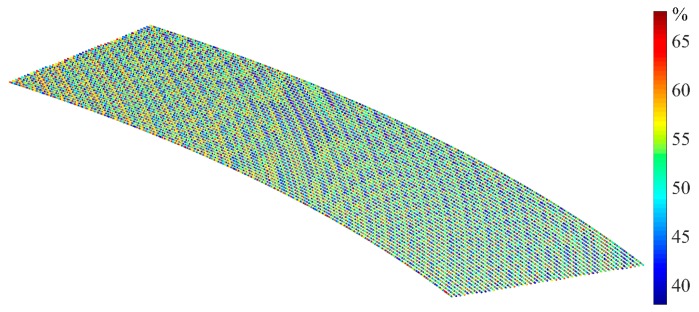
The improvement ratio in the compensation results compared to benchmark data.

Figure 11 is the comparison results, which shows that the measuring accuracy of the laser triangulation can be improved by at least 40%. This demonstrates that both the empirical compensation model in Equation (15) and the measurement suggestions proposed in Section 4 and Section 5 are feasible for use in engineering measurements. The distribution of the improvement percentage is confirmation that the degree of sensitivity of the optical paths of areas in which the curvatures change considerably is susceptible to yield error.

## 7. Conclusions

In this paper, we addressed the error compensation problems of depth measurement with laser triangulation probes. Through the analysis of geometrical optics under the existing oil film on the measuring surface, the essential factors affecting the measuring accuracy had been found, including the thickness and refractive index of oil film, and the theoretical error model associated with these internal parameters of oil film was developed to make the compensation procedure. Then by using an inclined model to evaluate the influence pattern of the probe’s position and orientation parameters (including the scan depth, incident angle and azimuth angle), we concluded that the scan depth has a relatively small impact when the measuring object is near the center of probe’s measurement range, whereas the effects of the incident angle and azimuth angle are more significant. To reduce their overall impact on the measuring accuracy, an empirical compensation model was established from the regression analyses. Suggestions were also proposed to reduce the impact of the filtering process, including considering both the filtering properties and the geometric features of the measurement target when selecting the filtering method.

Three kinds of oily substances and a chevron-corrugated plate were used to verify the effectiveness of both the proposed models and our measuring recommendations. Comparative results demonstrated that these compensation strategies for improving the measuring accuracy of the laser triangulation probe on the depth measurement are effective.

Notably, there is still room for future research. In this study, we did not consider other factors that might influence the measuring process, such as illumination, temperature, and mechanical vibrations. To improve the robustness of these methods and limit the variation in the results caused by external physical parameters, future research should focus on the development of an adaptive algorithm to implement a dynamic software compensation.

## Figures and Tables

**Table 1:** The main configuration parameters of the KEYENCE LK-H080 probe.

Parameter	Specifications
Installation mode	Diffuse reflection
Reference distance	80 mm
Measuring range	±18 mm
Beam diameter	70 μm
Linearity	±0.02% F.S. (Full Scale: F.S.; F.S. = 36 mm)
Repeatability	0.1 μm
Resolution	0.1 μm
Sampling frequency	2.55/5/10/20/50/100/200/500/1000 μs
Temperature characteristic	0.01% F.S./°C
Communication mode	RS232/USB/Ethernet
